# A bio-objects approach to biosecurity: the “mutant flu” controversy as a bio-objectification process

**DOI:** 10.3325/cmj.2013.54.592

**Published:** 2013-12

**Authors:** Jose A. Cañada

**Affiliations:** University of Helsinki, Department of Social Research, Unit of Social Psychology, Helsinki, Finland

The governance of biological emergencies has been an important issue during the last decade. Much policy work has been done on the topic, aiming at covering the necessities associated to bio-preparedness – ie, preparedness for biological threats. In this context, there is a lot to be said and understood by looking at emerging biological entities, such as new viruses or bacteria. Some of the most important international organizations worry about this issue: The North Atlantic Treaty Organization (NATO), World Health Organization (WHO), or European Union (EU) have specific sections specialized in dealing with this matter. Such concern has been fed by the expert opinion asserting that a big pandemic is imminent. This is based on several developments that have taken place during the last decades: the 80s saw the expansion of Ebola and HIV, and the 90s and 2000s witnessed the appearance of the Creutzfeld-Jacob’s disease, severe acute respiratory syndrome (SRAS), and different types of influenza viruses coming from other species, such as the avian influenza or the H1N1. These outbreaks, together with the Anthrax attacks that took place in the aftermath of 9/11 and the research on genetic engineering of viruses has led to a general state of fear of biological threats. Such events are characterized by a strong interaction between the social and the biological, between populations and viruses. I suggest that conceptualizing viruses as bio-objects will be helpful in our attempt to understand such interaction.

The bio-objects framework, which has been developed by the research network “Bio-objects and their Boundaries: Governing Matters at the Intersection of Society, Science and Politics” (1,2) has provided an interesting way of thinking about emerging biological entities. A number of analyses with different biological entities have already been reported in this same journal. MicroRNA’s (3), dried blood spots (4), genetically modified insects (5), transgenic food (6), or HeLa cells (7) serve as examples. Such variety of work shows the versatility of the concept. In an attempt to understand the dynamic processes that give raise and regulate emerging biological entities, the concept provides a theoretical and methodological tool useful to explore new fields related to biology and life sciences.

In this article I will claim that the bio-objects framework is a useful tool to connect some biological dimensions of biosecurity with the political and the social. The framework is beneficial when we aim at tracking and following biological entities that are controversial and subject to change. Controversies are common in the area of biosecurity, an area that embraces institutional entities situated in the intersection of science, economy, security, law, society, and politics. It is within this background that viruses need to be understood nowadays: as objects that transgress existing boundaries and classifications while their identity is continually challenged. They are, in that sense, suitable to be understood as bio-objects. I will try to illustrate these ideas using a recent controversy on the consequences of genetic engineering on Highly Pathogenic Avian Influenza (HPAI) A/H5N1, a virus that appeared in 1997 in Southeast Asia and that has been threatening to overcome the human/animal interface since then. By following research articles, pieces of news and reports, I have carried out an exploratory analysis that intends to understand viruses as immersed in a bio-objectification process.

## Social research on biosecurity

Three different sources are considered by biosecurity policymakers: a) natural outbreaks, b) bioterrorism, and c) laboratory accidents. What is interesting about this 3-fold interaction is that the three of them incorporate disparate rationalities coming from different sociopolitical practices. While natural outbreaks are usually a public health issue, bioterrorist attacks are a civil defense and national security concern. The former is in deep relation with medicine and epidemiology, the latter with military forms of thought. This interaction is deeply related to United States (US) politics where, during the last quarter of the 20th century, diseases that were approached exclusively as medical issues became an issue for national security too (8). The third rationality mentioned is the one of laboratory accidents, mainly represented by accidental releases. This is regulated through biosafety rules, which are guided by the WHO, leaving the responsibility scattered among scientists, research institutions, and funding organizations. This third rationality interacts with the two previous ones by incorporating a controversial dilemma: life sciences research entails threat and progress in the same activity.

**Figure 1:**
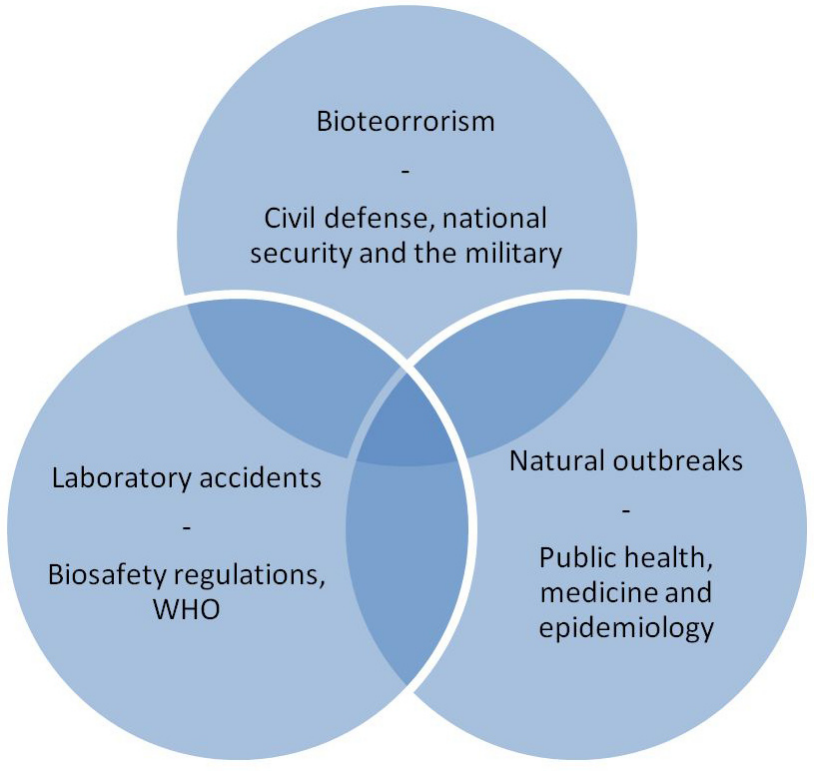
The three sources of threat for biosecurity policies and their respective rationalities.

The interaction of these three rationalities is also a source of controversy in the field of biosecurity as it integrates different institutions and disciplines that have a common interest but that have historically had different objectives. The emergence of an infectious agent in the population is handled through policies that fall under the umbrella of biosecurity, a wide term that enrolls many institutions that work in different political disciplines. This implicates that a given outbreak is understood under the light of military, health, and research rationalities at the same time (Figure 1).

Policies pick up this biological threat and are developed discursively in a state of uncertainty, risk, threat, and fear. These four ideas are in the core of any anticipation of a biological emergency. In that state of alert, a biological event is expected to happen, though we do not know when, where, or how. Threat and fear are instituted as the new normality (9) and biosecurity scenarios (10) are the only way to evaluate future events. This alert attitude needs to be maintained in order to be prepared to act in front of an emergency. A constant state of uncertainty brings in deep difficulties for governance processes. As future happenings are uncertain, effective policymaking becomes an extremely difficult task, if possible at all. In cases of sociotechnical controversy, the higher the stakes are, the less certainty is allowed (11). As the consequences of a biological event are hardly predictable but usually categorized as “devastating,” some experts and policymakers feel the need of preparing for any possibility, no matter what the probabilities of the event may be.

Under the effect of such discourses, biosecurity policies enter the field of bio-preparedness. In front of an unknown threat they adopt the perspective of what is called an “all hazards approach.” Countries need to get themselves ready for any kind of possible threat. Calculations and probabilities seem to be left out here: a distant possibility, because of its potential effect, is taken as a very close one. In this context, the logics of biosecurity move from prevention to precaution and to preparedness (12). The first one, prevention, linked to veterinary practices, relies on data of prevalence and incidence: it is a rationality of cases and propagation zones and knowing the enemy means being able to fight it. Precaution, on the other hand, pays attention to the limitations of knowledge. In such a case, the possibility of a trustable prediction is put in doubt, opening space for public deliberation. Finally, preparedness blurs knowledge and focuses directly on the potential consequences of an event. It is not that the new rationality erases the previous one, but it limits its sphere of application. The lack of knowledge in preparedness is dealt through scenario development and simulation, where fiction and reality meet (13). As fiction easily tends to overcome reality, the influence of fiction-based risk assessment is much more profound than knowledge-based risk assessment. Hence, policies and funding seem to shift their interest more toward preparedness than toward prevention.

Preparedness policies are built in a context characterized by the on-the-making processes that flood biosecurity controversies and are characterized by constant development. They are deeply rooted in the idea that preparedness is continuous (13); it is not seen as a final objective but as an ongoing process that can hardly be closed down. As these features are part of the most basic ideas laid down under such policies, they deploy what I call “standby policies.” These policies are designed to cover future events but they cannot be implemented unless such events happen. They are developed in a symbolic hypothetical context. Still, they have an actual effect in life governance while their formulation remains virtual (14).

Such discourses give meaning to the current state of affairs of research on emerging infectious diseases (EIDs). The controversy analyzed here cannot be understood as an isolated case, but as a consequence of decades of political, social, and scientific activity.

## The mutant flu controversy (15,16)

In September 2011, Ron Fouchier and his team, from the Erasmus Medical Center in the Netherlands, reported at the European Scientific Working Group on Influenza’s conference in Malta having pushed certain mutations in the long time known A/H5N1 Influenza virus. These mutations made the virus able to be transmitted among ferrets, the most used animal model in human Influenza research. Concurrently, Yoshihiro Kawaoka, who runs two biolaboratories, one at the University of Wisconsin-Madison and another one at the University of Tokyo, carried out similar research with similar conclusions. Both researches were funded by the US government. Papers reporting on the results were sent to leading journals: Fouchier’s to *Science* and Kawaoka’s to *Nature*. But before publication, the National Science Advisory Board for Biosecurity (NSABB), the US institution competent in the matter, reviewed both papers to evaluate their implications related to dual-use research concerns. The papers were evaluated during October 2011 and a decision was made public in December: the papers would be published with redaction of the methodological design in order to avoid potential misuse of the mutant virus. Access to all the details of the research would be provided to certain authorized researchers. The publication was scheduled to take place during March 2012. As part of the controversy, a 60-day moratorium on HPAI H5N1 research was declared on January 2012, which finally lasted more than one year, until February 2013. The objective of such moratorium was “to provide time to explain the public health benefits of this work, to describe the measures in place to minimize possible risks, and to enable organizations and governments around the world to review their policies” (17). During that time, in February 2012, a new meeting between WHO and NSABB members took place. The result of such meeting was that both papers needed to be published fully. The arguments that led to this decision were the need to stimulate public health research and the difficulty on deciding who would and would not have access to the non-redacted material. A final meeting between the NSABB and the authors took place in March 2012 in order to revise the final drafts. At last, by June 22, 2012, both papers had been published (18,19).

The controversy was mainly fed by the discussion on the potential risks and benefits of H5N1 gain-of-function research. So, the first questions asked were related to how dangerous or beneficial the research actually was. On the one hand, the ability to make H5N1 transmissible in mammals could be used by bioterrorists and the fail of biosafety techniques could provoke an accidental release of the virus. On the other hand, knowing how the virus might develop can help to assess the threat posed by viruses sampled from wild or farm animals and human confirmed cases. Another source of problems that fed the controversy was the legal and political framework that surrounded it. US law did not allow redacting the research partly: it had to be published fully or classified. Also, politically, it raised problems at the international level. The Pandemic Influenza Preparedness (PIP) Framework, run by the WHO, is a program that engages many countries and it is considered a basic tool for Influenza preparedness. Not sharing this information with the rest of the members of the PIP could be seen as problematic, motivating some countries to fall off the program (as Indonesia had done in front of another polemical situation regarding the fabrication of Influenza vaccines in 2006) (20).

The controversy was solved/silenced thanks to the final decision about how the research articles should be published. But consequences were to follow. US carried out policy changes that now allow government officers to review and monitor biological research implicating any pathogen present on the list of 15 biological entities of potential dual-use concern. US government is also allowed, under such policy changes, to modify the methodological design and the conduct of US funded research.

## The A/H5N1 Influenza virus as a bio-object: the bio-objectification process

The A/H5N1 Influenza was first detected among humans in 1997. Since then, the virus has been identified as causing zoonotic disease. In the period from the beginning of 2003 to October 8, 2013, 641 laboratory cases were reported to the WHO, 380 of them being lethal (21). H5N1 has been a pandemic threat for about 16 years now. Its stability as an object has been subjected to the emergence of new mutations, to peaks in its contagiousness on humans or animals, and to public controversies. The so-called “mutant flu” controversy has been one of those moments when stability becomes compromised and the bio-objectification process becomes visible and suitable to be analyzed.

As we take a closer look to the experiments carried out by Fouchier and Kawaoka, the identity held by A/H5N1 is already fragmented as different samples need to be separately identified. For example, Fouchier experimented with the virus A/Indonesia/5/2005 and Kawaoka with the A/Vietnam/1203/2004. These names serve already as identifier that stabilizes the sample (22), as an object susceptible of being collected, stored, and shipped. Being the samples already linked to the time and place of their collection, mutation in the laboratory implicates a new identity already: the virus result of biotechnological labor will differ from the original sample. We need to also acknowledge that such identities are not equally relevant for everyone: such fragmentation of the main H5N1 identity is relevant for experts but not entirely for other milieus. For example, most journalists, lay people, and politicians will keep referring to the virus using the name H5N1.

The bio-objectification process starts to be evident inside the controversy as the sample is transported from a biobank in Hong Kong to a biolaboratory in Amsterdam, in the case of Fouchier, and from Vietnam to the US, in the case of Kawaoka. After such process, biotechnological labor is carried out. Both samples are submitted to modifications in order to potentiate their capability to be transmitted among mammals. In the case of Fouchier, by genetically modifying the virus using site-directed mutagenesis and subsequent serial passage in ferrets, the virus was able to be transmitted among ferrets. Furthermore, such material changes also affected the pathogenicity of the virus. Besides the ability of transmission, the fatality was drastically reduced and none of the recipient ferrets died after airborne infection with the mutant A/H5N1 viruses. In the case of Kawaoka, the team identified a reassortant H5 HA/H1N1 virus. This was a combination of H5N1 and an H1N1 sample from the 2009 pandemic influenza outbreak. Such reassortant was capable of droplet transmission in a ferret model. Kawaoka’s virus replicated successfully among ferrets but was not highly pathogenic and did not cause mortality. The changes on their capacities are central to understand the controversy. The previous point of concern for biosecurity was the high pathogenicity of the A/H5N1 Influenza, and its ability to spread was the only thing keeping the virus out from becoming a pandemic (although it was considered a pandemic threat). Both mutated viruses incorporate the ability to be transmitted among humans (inferred through an animal model), but lose its high pathogenicity. At this point, the materiality and capacities of the virus have changed but its identity keeps being the same for a big part of the society, including politicians and regulators.

**Figure 2:**
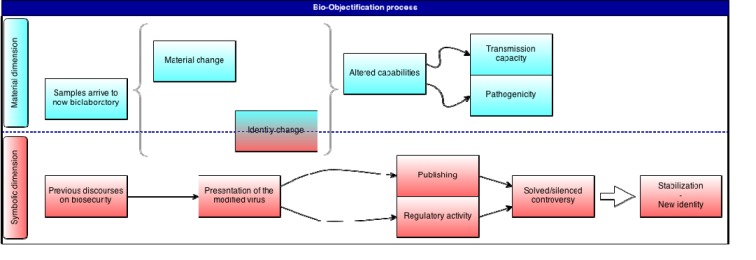
The bio-objectification process.

Subsequently, regulation – a process inside the symbolic dimension of the bio-objectification process – is required as the new capabilities and materiality do not totally fit with the known identity of the virus. In this new regulatory activity, NSABB, WHO, and the journals *Science* and *Nature*, as well as mass media, attributed a series of features that will configure the identity of the object: mutant, deadly, or lethal are adjectives that became attached to the new identity of the virus. Such process has been deeply conditioned by the material changes but it is not until regulatory agents enter the scene that the identity change starts to be evident, as the material change process was when the samples started to move. Material change is situated in the material dimension of the process, while identity change needs to be understood in the interface between the material and the symbolic dimension (Figure 2). Both dimensions are inseparable as they are necessarily intertwined, but they do not share actors, practices, and spaces during the whole process. The final decision and regulation of the data related to the new agent allows the controversy to be solved/silenced, conferring some stability to the bio-object and its identity.

But what are the consequences of this process? How does society apprehend such changes? The first interesting consequence of bio-objectification processes is the challenging of existing classifications and boundaries. Laboratory/nature becomes a relevant dichotomy for the configuration of the biological object. Through laboratory practices, nature is anticipated. The previously uncertain event of a natural mutation of a H5N1 Influenza virus is not only artificially predicted but also artificially enacted (the fictionalized prediction of a pandemic is, in a way, voluntarily pursued). Species boundaries are clearly another set of barriers that is challenged by the controversy. While the use of animal models, such as ferrets, is nothing new in biological research, the genetic engineering of the virus reminds us of the mobile barrier between epidemic, epizootic, and zoonotic diseases. A third category of challenged boundaries is the one of disciplines. As the controversy develops, the new virus enters, discursively, more and more disciplinary areas. The challenges for the researcher are beyond microbiology. They need to answer in front of the press, committees, boards, etc. The virus has entered the space of politics, law, international relationships, and security.

A second interesting consequence of such modifications is that the values attached to A/H5N1, as well as its own value as an object, are reevaluated. The potential identities of the virus imply new forms of understanding it as a threat. While before it was classified as a natural threat, now it is classified as a human-made threat – therefore avoidable to some extent. Value creation is always in relation to the interactions of the virus with other social actors. In the proper hands, the virus will acquire the proper value – ie, a positive value. Researchers will use it for public health benefit. In the wrong hands, it will acquire negative value as terrorists could use it for nefarious purposes. But it is important to acknowledge the uncertainty of such changes. The abilities to carry out genetic engineering are not so easy to learn and, on the other hand, laboratory accidents are not only dependent on the good will of researchers. So, whether the engineered virus will become a great opportunity to tackle the pandemic influenza threat or a great threat for the entire humanity remains unknown. This new potential value, still uncertain, feeds the rationality of preparedness.

## Conclusions

Thinking of biological controversies in terms of bio-objectification allows us to reflect on materiality and meaning as processes that, far from being stable, are constantly on-the-making. The eventual and transitory stability of biological entities is always linked to broader social processes that escape scientific practice. The attention paid by the bio-objects framework to political and, more precisely, regulatory practices, is crucial to understand the stabilization of bio-objects submitted to processes of biotechnologization and their identification processes. Furthermore, the bio-objects framework seems to be useful in order to analyze the most molecular dimensions of biosecurity, having its starting point in the technical labor carried out in microbiology laboratories. This way, it helps connecting the practical dimensions attached to the practice of microbiology to the political dimensions attached to policymaking in biosecurity.

## References

[1] Metzler I, Webster A (2011). Bio-objects and their boundaries: governing matters at the intersection of society, politics, and science.. Croat Med J.

[2] Holmberg T, Schwennesen N, Webster A (2011). Bio-objects and the bio-objectification process.. Croat Med J.

[3] Chrupek M, Siipi H, Martinelli L (2012). Bio-objects as “boundary crawlers:” the case of microRNAs.. Croat Med J.

[4] Douglas CMW, van El CG, Faulkner A, Cornel MC (2012). Governing biological material at the intersection of care and research: the use of dried blood spots for biobanking.. Croat Med J.

[5] Reis-Castro L (2012). Genetically modified insects as a public health tool: discussing the different bio-objectification within genetic strategies.. Croat Med J.

[6] Martinelli L, Karbarz M, Siipi H (2013). Science, safety, and trust: the case of transgenic food.. Croat Med J.

[7] Svalastog AL, Martinelli L (2013). Representing life as opposed to being: the bio-objectification process of the HeLa cells and its relation to personalized medicine.. Croat Med J.

[8] Lakoff A (2008). The generic biothreat, or, how we became unprepared.. Cult Anthropol.

[9] Massumi B (2005). Fear (The Spectrum Said).. Positions (Durh N C).

[10] Collier SJ, Lakoff A, Rabinow P (2004). Biosecurity towards an anthropology of the contemporary.. Anthropol Today.

[11] Sarewitz D (2004). How science makes environmental controversies worse.. Environ Sci Policy.

[12] Keck F. From Mad Cow disease to bird flu: transformations of food safety in France. In: Lakoff A, Collier S, editors. Biosecurity interventions: global health and security in question (p. 195). New York: Columbia University Press; 2008. p. 195-225.

[13] Lakoff A (2007). Preparing for the next emergency.. Public Cult.

[14] Bryant LR (2004). Politics of the virtual.. J Psychoanal Cult Soc.

[15] Gronvall GK. H5N1: a case study for dual-use research. New York: Council on Foreign Relationships; 2013.

[16] Lakoff A (2012). The risks of preparedness: mutant bird flu.. Public Cult.

[17] Fouchier RAM, García-Sastre A, Kawaoka Y, Barclay WS, Bouvier NM, Brown IH (2013). Transmission studies resume for avian flu.. Science.

[18] Herfst S, Schrauwen EJA, Linster M, Chutinimitkul S, de Wit E, Munster VJ (2012). Airborne transmission of influenza a/h5n1 virus between ferrets.. Science.

[19] Imai M, Watanabe T, Hatta M, Das SC, Ozawa M, Shinya K (2012). Experimental adaptation of an influenza H5 HA confers respiratory droplet transmission to a reassortant H5 HA/H1N1 virus in ferrets.. Nature.

[20] Fidler DP (2008). Influenza virus samples, international law, and global health diplomacy.. Emerg Infect Dis.

[21] World Health Organization. Cumulative number of confirmed human cases for avian influenza A (H5N1) reported to WHO, 2003-2013. Geneva: World Health Organization; 2013. Available from: http://www.who.int/influenza/human_animal_interface/EN_GIP_20131008CumulativeNumberH5N1cases.pdf*.* Accessed: December 22, 2013.

[22] Butler D (2008). Politically correct names given to flu viruses.. Nature.

